# Editorial Comment: Severity of Lower Urinary Tract Symptoms Predicts Neurologic Quality of Life in Patients With Multiple Sclerosis

**DOI:** 10.1590/S1677-5538.IBJU.2025.9911

**Published:** 2025-04-15

**Authors:** Lily Kong, Aleksandar C. Blubaum, Stephen A. Blakely, Augusto A. Miravalle, Brian J. Flynn

**Affiliations:** 1 University of Colorado Anschutz Medical Campus Department of Surgery, Division of Urology Aurora Colorado United States Department of Surgery, Division of Urology, University of Colorado Anschutz Medical Campus, Aurora, Colorado; 2 University of Oklahoma Health Sciences Center Department of Urology Oklahoma City Oklahoma United States Department of Urology, University of Oklahoma Health Sciences Center, Oklahoma City, Oklahoma, Oklahoma City, Oklahoma;; 3 SUNY Upstate Medical University Department of Urology Syracuse New York United States Department of Urology, SUNY Upstate Medical University, Syracuse, New York;; 4 University of Colorado Anschutz Medical Campus Department of Neurology Aurora Colorado United States Department of Neurology, University of Colorado Anschutz Medical Campus, Aurora, Colorado

JU Open Plus 2(9):e00094, September 2024.

DOI: 10.1097/JU9.0000000000000203 |

## COMMENT

The study published by Kong et al. aimed to define the incidence of lower urinary tract symptoms (LUTS) in patients with multiple sclerosis (MS) and their effect on neurologic quality of life. LUTS were assessed through validated questionnaires, such as the Bladder Control Scale (BLCS) from the Multiple Sclerosis Quality-of-Life Inventory (MSQLI) and overall Quality of Life in Neurological Disorders (NeuroQOL). Ninety-three percent of the patients described the presence of at least 1 LUTS. The most common symptoms were urgency (84%), frequency (69%), incontinence (54%), and retention (38%). Seventy-two (79%) patients reported that LUTS negatively affected urinary quality of life.

Multiple Sclerosis is a chronic neurological disease that may include signs and/or symptoms such as muscle weakness, changes in gait and balance, pain, changes in language and communication, and urinary tract symptoms. Additionally, psychosocial issues, such as depression, anxiety, social stigmas, high costs for maintaining healthcare, limitations in social activities, loss of independence or even social isolation. Therefore, a comprehensive assessment of MS is recommended and must include targeted medical history and questionnaires, which allow not only a proper classification of LUTS, but also their severity and impact on social activities. Appropriate management of neurogenic lower urinary tract dysfunction requires a multimodal and interdisciplinary approach to effectively improve symptoms. Also, rehabilitation or conservative treatment plays a very important role in this field, comprising of behavioral modifications, pharmacological treatment, intermittent catheterization, pelvic floor muscle training, as well as tibial nerve stimulation (1, 2). Advanced treatments such as botulinum toxin bladder injections and sacral neurostimulation may be considered for selected MS patients as well (3).
